# Efficacy of Non-pharmacologic Auxiliary Treatments in Improving Defecation Function in Children With Chronic Idiopathic Constipation: A Systematic Review and Network Meta-analysis

**DOI:** 10.3389/fped.2021.667225

**Published:** 2021-04-27

**Authors:** Jie Tang, Huijuan Li, Weibing Tang

**Affiliations:** ^1^Department of Pediatric Surgery, Children's Hospital of Nanjing Medical University, Nanjing, China; ^2^Department of Neonatology, Children's Hospital of Nanjing Medical University, Nanjing, China

**Keywords:** children, chronic idiopathic constipation, network meta-analysis, auxiliary therapies, non-pharmacologic treatments

## Abstract

**Background:** Non-pharmacologic auxiliary treatments have been considered crucial therapies for treating chronic idiopathic constipation (CIC) during the past decades worldwide. Several treatment patterns are available, but their relative efficacy is obscure because there are no head-to-head randomized controlled trials, especially in children. We conducted this network meta-analysis to evalute the effectiveness of these therapies in improving defecation function based on their direct comparisons with standard medical care.

**Methods:** Medline, Embase, and Cochrane Central were searched for randomized controlled trials (RCTs) published in English from inception to October 2020, assessing the efficacy of auxiliary therapies (behavior therapy, physiotherapy, biofeedback, or anorectal manometry) in children with CIC. We extracted data for endpoints, risk of bias, and evidence quality. Eligible studies in the meta-analysis reported the data of a dichotomous assessment of overall response to treatment (response or not) or defecation frequency per week after treatment. The hierarchical Bayesian network meta-analysis was used in the study. We chose a conservative methodology, random effects model, to pool data which could handle the heterogeneity well. The relative risk (RR) with 95% confidence intervals (CIs) was calculated for dichotomous outcomes. For continuous results, weighted mean difference (WMD) with related CIs was calculated. The included treatments were ranked to define the probability of being the best treatment.

**Results:** Seven RCTs (838 patients) met inclusion and endpoint criteria. Based on an endpoint of the absence of constipation (Rome criteria) with laxatives allowed, physiotherapy plus standard medical care (SMC) had the highest probability (84%) to bethe most effective therapy. When the treatment response was defined as an absence of constipation with not laxatives allowed, biofeedback plus SMC ranked first (probability 52%). Physiotherapy plus SMC ranked first when the endpoint was based on defecation frequency per week with laxatives allowed (probability 86%).

**Conclusion:** Almost all auxiliary therapies are effective complementary therapies for treating CIC, but they needed to be used simultaneously with SMC. Nevertheless, because of the small number of eligible studies and their small sample sizes, the differences in treatment duration and the endpoints, large sample RCTs with long-term follow-up are required for further investigation.

## Introduction

Chronic idiopathic constipation (CIC) is a common pediatric gastrointestinal disorder worldwide (1, 2). Patients with CIC always suffer from abdominal pain, painful bowel movements, large stools, and fecal incontinence (2, 3). At present, the standard medical care of functional constipation contains dietary adjustment, toilet training, reassurance, education, and the use of laxatives (2, 4, 5). It was reported that 50% of the children receiving standard medical care (SMC) still have symptoms after several years and that the complaints might persist till adulthood (6, 7). Studies in the USA reported that constipation caused millions of medical visits and substantial medical costs (8, 9).

Some non-pharmacologic auxiliary treatments have been deeply investigated during past decades, and the application of these therapies has significantly increased, such as behavior therapy, physiotherapy, biofeedback therapy, etc. (10–12). However, these studies focused on whether these auxiliary treatments had better effects and equal safety compared with standard medical care. There were no head-to-head randomized controlled trials to evaluate the relative efficacy of these auxiliary therapies, especially in children. Besides, due to economic development, social culture and other factors, not all countries and regions could provide all auxiliary therapies. The current information could not guide clinicians and policemakers to choose relatively better treatments according to local conditions to help these patients. With an increasing interest in these auxiliary treatments worldwide, we determined to conduct this network meta-analysis to compare these auxiliary treatments in efficacy of improving defecation function. We hope that this research cloud provide evidence and new insight on chronic idiopathic constipation treatment in children.

## Methods

According to the Cochrane Handbook for Systematic Reviews and Meta-Analyses guidelines (13), this network meta-analysis was performed. The Preferred Reporting Items for Systematic Reviews and Meta-Analyses (PRISMA) statement was adopted to guide the report of final results (14). The study followed a pre-specified study protocol registered in PROSPERO (CRD42020214699).

### Search

A database search was performed in October 2020, using Medline, Embase, and Cochrane Central for treating chronic idiopathic constipation in children. A comprehensive search strategy was established based on free-text terms, keywords, and medical subject heading (MeSH) terms, including constipation, children, and randomized controlled trial. Multiple different combinations of these terms were conducted by using “and/or.” All abstracts obtained according to the search strategy were independently evaluated by two investigators (TJ and LHJ) to screen out the studies matching the inclusion. For studies that might meet the inclusion criteria, full-text articles were requested. All the studies were independently determined by two researchers (TJ and LHJ) based on well-defined inclusion criteria. The conflicts were resolved after discussion between the researchers and a third researcher (TWB) with content expertise.

### Inclusion Criteria

This meta-analysis was required to be conducted in randomized controlled trials of non-pharmacologic therapies for CIC treatment in children (<18 years). The controls were set as the SMC for treating CIC. The definition of CIC in this meta-analysis was based on the diagnosis of clinicians or the specific symptom-based criteria (e.g., the Rome criteria). Studies were excluded if the symptoms of constipation were complications of surgeries or other diseases, as well as drug-induced constipation.

### Outcome Assessment

Curative rate (response to therapy) and defecation frequency were endpoints analyzed in this meta-analysis. In the eligible studies, the curative rate was defined as an absence of constipation with or without laxatives allowed. Defecation frequency was defined as defecation frequency per week with laxatives allowed. Because all of the above three have important clinical significance and are not included in each of the eligible studies, we did the subgroup meta-analysis of the three outcomes separately.

### Data Extraction and Management

Two independent investigators (TJ and LHJ) performed data extraction from the eligible studies. Data were extracted as numbers of responders and non-responders or mean ± SD for defecation frequency per week. We also collected data about characteristics of the eligible studies, such as study center location (by countries), number, age and gender distribution of subjects, criteria for diagnosing chronic idiopathic constipation, and study endpoints. Two investigators independently extracted relevant information such as allocation concealment, blinded model, and the method of follow-up analysis to evaluate the study quality of the study.

### Statistical Analysis

Data of intention-to-treat was used in this study. Bayesian network meta-analysis was conducted for each clinical endpoint by random effects consistency models (15–17). In the network meta-analysis, each eligible study contains similar and exchangeable (that is, transitivity) treatment effects (18). The network meta-analysis could combine direct or indirect evidence to produce relative treatment effect estimates (16, 19). We chose a conservative methodology, random effects models, to pool data that could handle the heterogeneity well (18, 20). The relative risk (RR) with 95% confidence intervals (CIs) was calculated for dichotomous outcomes. For continuous outcomes, weighted mean difference (WMD) with related CIs was calculated. The network meta-analysis was conducted using the ADDIS software v1.16.8. Models were computed using four chains with over-dispersed initial values, with Gibbs sampling based on 50,000 iterations after a burn-in phase of 10,000 in iterations. Network meta-analysis results usually give a more precise estimate than standard, pairwise analysis and can rank treatment to guide clinical decisions. The included treatments were ranked to define the probability of being the best treatment (20–22).

We assessed statistical heterogeneity using the *I*^2^-statistic. The values were representing mild (<25%), moderate (25–75%), and severe heterogeneity (>75%) (23).

### Assessment of Risk of Bias

Risks of bias were assessed using Cochrane Handbook for Assessing the Risk of Bias. Two investigators independently evaluated the random sequence generation, allocation concealment, blinding of participants and investigators, blinding of outcome assessment, incomplete outcome data, selective reporting, and other biases. Due to the small number of studies included in this meta-analysis, it was hard to assess potential publication bias.

## Results

### Systematic Review and Qualitative Assessment

**Figure 1:**
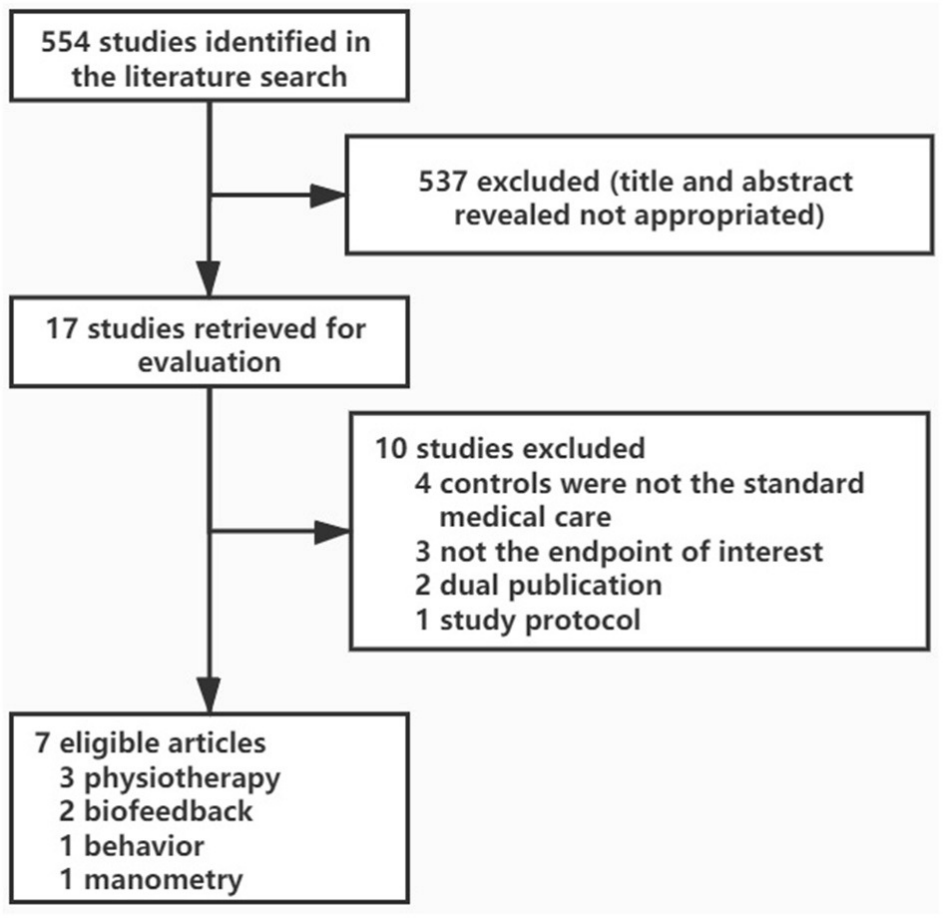
Study selection.

In total, 573 citations were generated using the search strategy. After a preliminary evaluation, 17 of them appeared to be relevant to this network meta-analysis, and subsequent further assessment was carried out. Of these studies, 10 were excluded (Figure 1). We included 7 eligible articles reporting 7 clinical trials of interest clinical outcomes, including an absence of constipation with laxatives allowed, absence of constipation with laxatives not allowed, and defecation frequency per week. All eligible studies' characteristics were shown in Table 1. These studies contained 838 patients allocated to SMC or non-pharmacologic auxiliary therapies or non-pharmacologic auxiliary therapies plus SMC. Since the three clinical outcomes could not be combined for analysis, we did the subgroup meta-analysis of the three endpoints separately. The risk of bias is reported in Supplementary Figure 1. Although not all studies reported a proper intention-to-treat analysis, we could extract the data from their articles and use them for further research. No studies conducted direct comparisons of one auxiliary therapy vs. another. As a result, an indirect evidence network meta-analysis relative to the comparison with SMC effects was made to evaluate the efficacy of several non-pharmacologic auxiliary treatments.

**Table 1:** Study characteristics.

**References**	**Country and number of centers**	**Endpoints used to define symptom improvement following therapy**	**Total number of patients**	**Gender, female %**	**Age (years)**	**Number of patients assigned to intervention**	**Number of patients assigned to control**	**Constipation criteria**
1. van Dijk et al. (10)	Netherlands, one site	Treatment was considered successful if patients achieved a defecation frequency of ≥3 times per week and a fecal incontinence frequency of ≤1 time per 2 weeks, irrespective of laxatives use.	134	58/134 (43.2%)	6.9 ± 2.5 vs. 6.5 ± 2.1	67 patients; Behavior therapy.	67 patients; Standard medical care.	Patients had to meet at least 2 of 4 criteria: defecation frequency <3 times per week, fecal incontinence ≥2 times per week, passage of large amounts of stool at least once every 7–30 days (large enough to clog the toilet), or a palpable abdominal or rectal fecal mass.
2. van Engelenburg-van Lonkhuyzen et al. (11)	Netherlands, one site	The absence of functional constipation according to the 6 Rome III criteria. This meant meeting 1 or fewer of the 6 Rome III criteria, irrespective of laxatives use.	53	29/53 (55%)	5–15 years	26 patients; physiotherapy intervention plus standard medical care.	27 patients; standard medical care training.	Rome III
3. van der Plas et al. (12)	Netherlands, one site	Treatment was considered successful if the patients achieved three or more bowel movements per week and <2 soiling or encopresis episodes per month while not receiving laxatives for 4 weeks.	192	36/192 (34%)	8.0 (5–16)	98 patients; Five biofeedback training sessions plus standard medical care.	94 patients; Standard medical care: laxatives, dietary, toilet training, and maintenance of a diary of bowel habits.	They had to fulfill at least two of four criteria for pediatric constipation and were included if they had been treated medically for at least 1 month before randomization. ① stool frequency <3 per week; ② two or more soiling and/or encopresis episodes per week; ③ periodic passage of very large amounts of stool at least once every 7–30 days; or ④ a palpable abdominal or rectal mass.
4. van Summeren et al. (24)	Netherlands, 5 sites	Defined as the absence of functional constipation (Rome III).	134	82/134 (61%)	7.6 ± 3.5 years	67 patients; physiotherapy plus standard medical care.	67 patients; standard medical care.	Rome III
5. Loening-Baucke (25)	USA	Patients were considered to have recovered from chronic constipation and encopresis if they met the following criteria: ≥3 bowel movements per week and ≤2 soiling episodes per month while not receiving laxatives for 4 weeks.	41	10/41 (24.3%)	5–16 years	22 patients; biofeedback treatment plus standard medical care.	19 patients; standard medical care.	If they had ≥2 soiling episodes per week and evidence of a huge amount of fecal material in the rectal ampulla at rectal examination.
6. van Ginkel et al. (26)	Netherlands, one site	Successful treatment was defined as a defecation frequency of 3 or more per week and fewer than 1 soiling/encopresis episode per 2 weeks and no use of laxatives.	212	69/212 (33%)	5-17 years	97 patients; 2 manometry sessions plus standard medical care.	115 patients; standard medical care.	Subjects had to fulfill at least 2 of 4 following criteria: (1) stool frequency fewer than 3 per week; (2) 2 or more soiling and/or encopresis episodes per week; (3) periodic passage of very large amounts of stool every; (4) a palpable abdominal or rectal fecal mass.
7. Silva CAG et al (27)	Brasil, one site	Defecation frequency per week with laxatives allowed	72	42/72 (58%)	4–18 years	36 patients; physiotherapy plus standard medical care.	36 patients; standard medical care.	Rome III

### Bayesian Network Meta-Analysis

**Figure 2:**
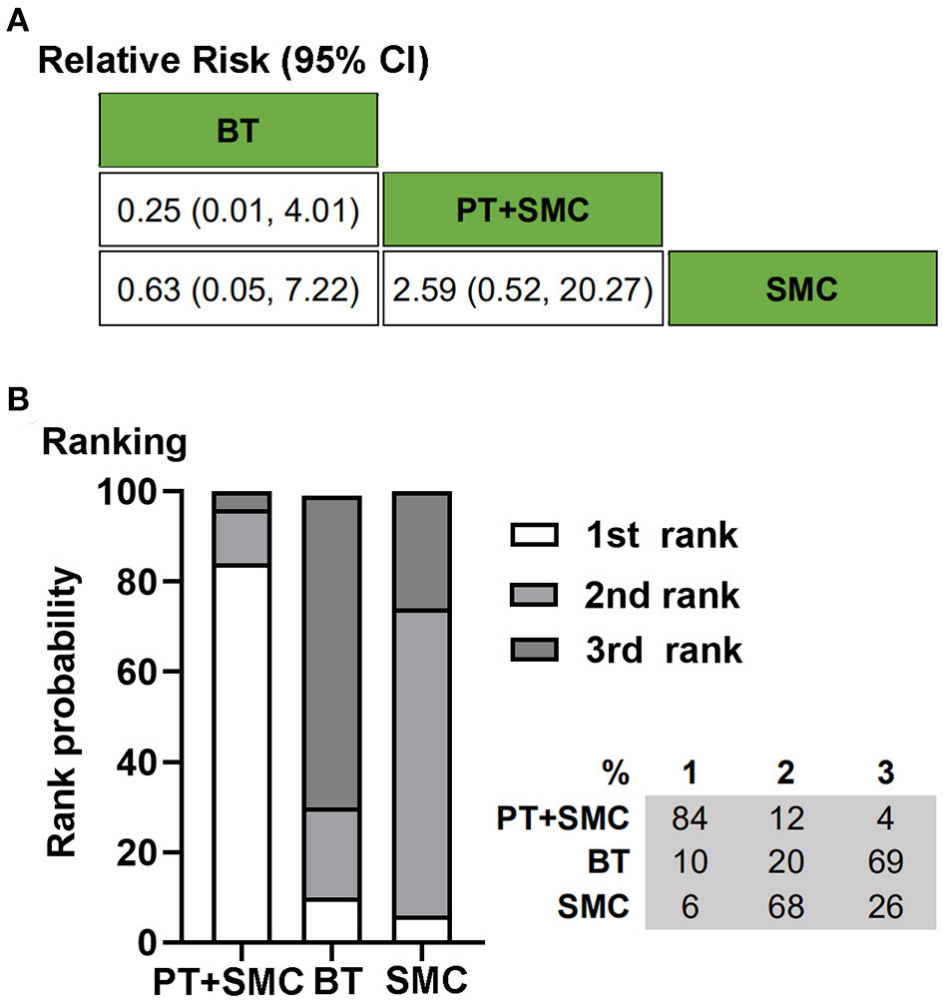
Bayesian network meta-analysis on the absence of constipation with laxatives allowed. **(A)** Data are relative risk (95% confidence interval, CI). Comparisons, column vs. row, should be read from left to right. **(B)** Corresponding rank probabilities of treatments. BT, Behavior therapy; PT + SMC, Physiotherapy plus Standard medical care; SMC, Standard medical care.

Three RCTs, including 321 children, reported data for the absence of constipation with laxatives allowed (10, 11, 24). One hundred and sixty (50%) patients were randomly assigned to behavior therapy or physiotherapy plus SMC. In this meta-analysis, physiotherapy was defined as the exercises for defecation-related muscles and training for sensory integration. Concerning the endpoint of the absence of constipation with laxatives allowed, physiotherapy could increase the success rate of the treatment on the basis of SMC. The results showed that SMC was better than behavior therapy alone. Treatment ranking indicated that physiotherapy plus SMC had a higher probability (84%) of being the best therapy (Figure 2).

**Figure 3:**
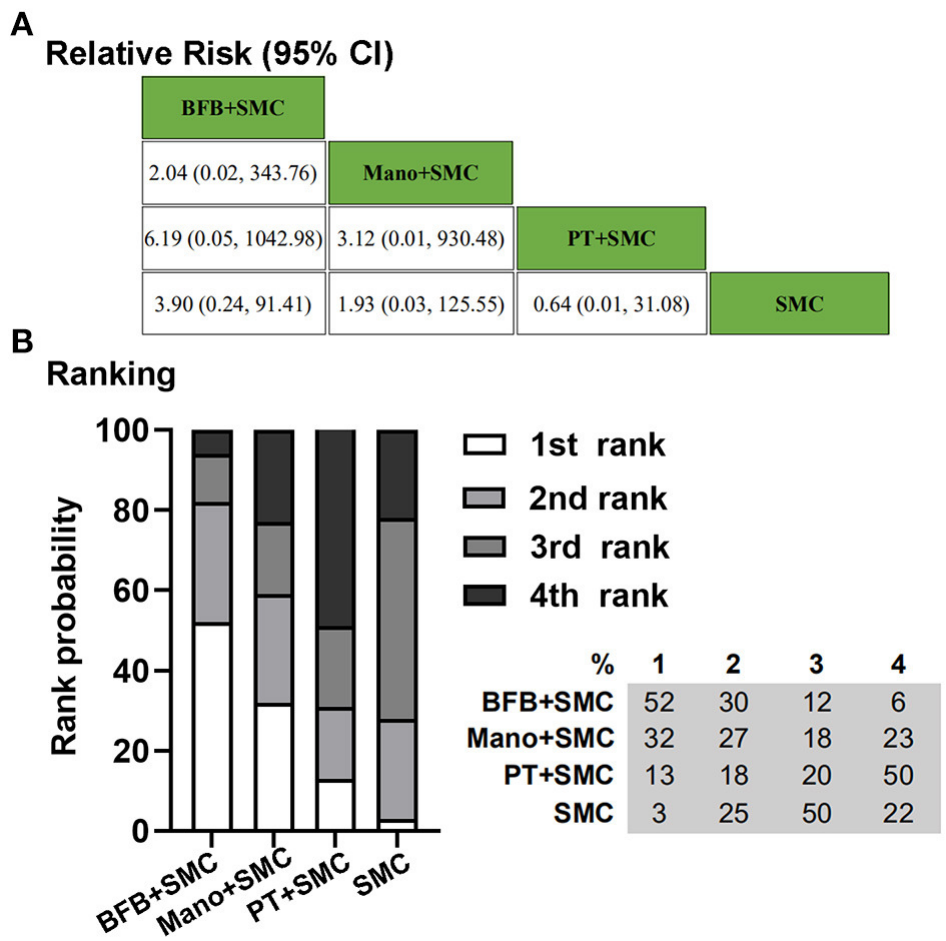
Bayesian network meta-analysis on the absence of constipation with laxatives not allowed. **(A)** Data are relative risk (95% CI). Comparisons, column vs. row, should be read from left to right. **(B)** Corresponding rank probabilities of treatments. BFB + SMC, Biofeedback plus Standard medical care; Mano + SMC, Anorectal manometry plus Standard medical care; PT + SMC, Physiotherapy plus Standard medical care; SMC, Standard medical care.

Four RCTs, including 579 children, reported data for the absence of constipation with laxatives not allowed (12, 24–26). Two hundred and eighty-four patients were randomly assigned to biofeedback plus SMC, anorectal manometry plus SMC, or physiotherapy plus SMC. With respect to the clinical outcome, absence of constipation with laxatives not allowed, all three adjuvant therapies could better affect the treatment of chronic idiopathic constipation based on SMC. Treatment ranking indicated that biofeedback plus SMC had a higher probability (52%) of being the best therapy (Figure 3).

**Figure 4:**
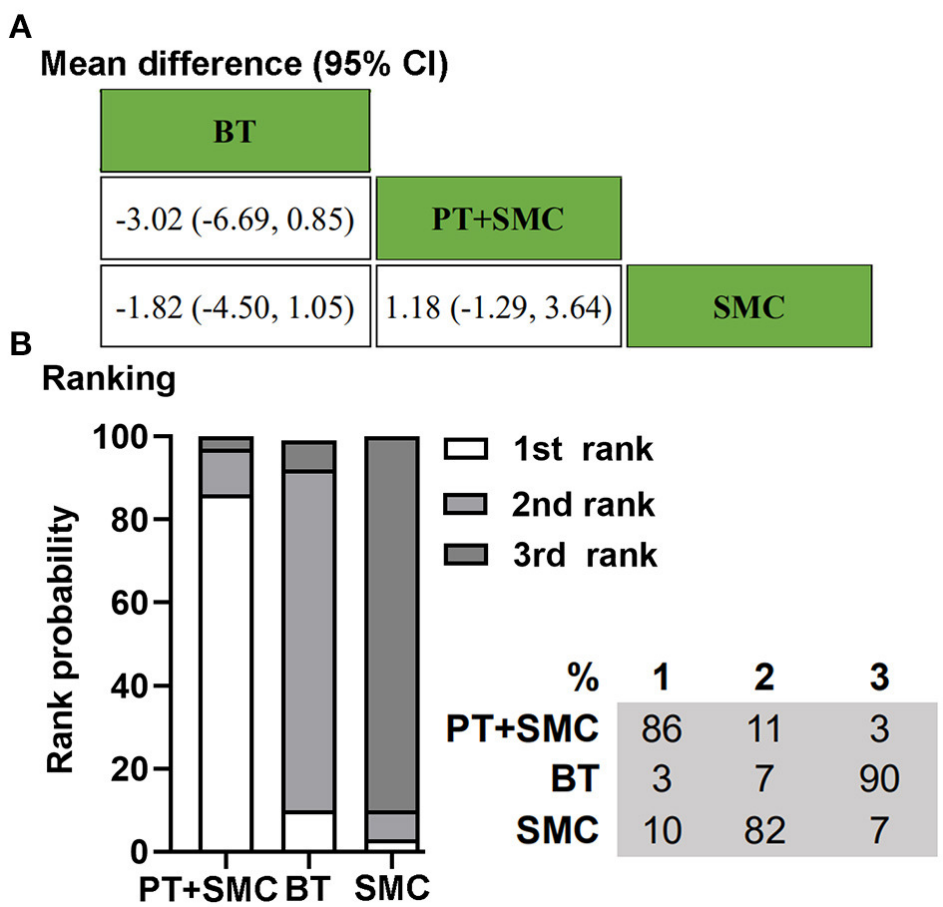
Bayesian network meta-analysis on defecation frequency per week with laxatives allowed. **(A)** Data are the mean difference (95% CI). Comparisons, column vs. row, should be read from left to right. **(B)** Corresponding rank probabilities of treatments. BT, Behavior therapy; PT + SMC, Physiotherapy plus Standard medical care; SMC, Standard medical care.

Two RCTs, including 346 children, reported data for defecation frequency per week with laxatives allowed (10, 27). One hundred and sixty-four patients were randomly assigned to behavior therapy or physiotherapy plus SMC. For the clinical outcome, defecation frequency per week with laxatives allowed, physiotherapy could play an auxiliary treatment effect based on SMC. Behavior therapy alone is not as effective as SMC. Treatment ranking indicated that physiotherapy plus SMC had a higher probability (86%) of being the best therapy (Figure 4).

All models converged adequately. Heterogeneity (global *I*^2^) was moderate for the absence of constipation with laxatives allowed and absence of constipation with laxatives not allowed, high for defecation frequency per week (90.3%) (Supplementary Figure 2). Model fitting was compared using the deviance information criterion and shown to be similar.

## Discussion

The network meta-analysis showed that non-pharmacologic therapies could improve clinical outcomes based on SMC. However, SMC was still a better choice than non-pharmacologic treatment alone for the treatment of constipation. As available studies were limited, and the included studies contained three different endpoints, we could not make an overall network analysis. As we know, the use of laxatives is an essential factor affecting the clinical outcome. we analyzed the three different endpoints, absence of constipation with laxatives allowed, defecation frequency per week with laxatives allowed, and absence of constipation with laxatives not allowed. For the two “laxatives allowed” related endpoints, we compared three treatments, behavior therapy, SMC, and physiotherapy plus SMC. Physiotherapy plus SMC ranked first, whether based on the treatment success rate or the frequency of defecations. Concerning the “laxatives not allowed” related endpoint, we compared four treatments, biofeedback plus SMC, anorectal manometry plus SMC, physiotherapy plus SMC, and SMC. The biofeedback therapy plus SMC ranked first, while physiotherapy plus SMC was only better than SMC. According to the results, we found that SMC was better than a non-pharmacologic therapy used alone. However, based on SMC, any additional auxiliary treatments could increase benefits. Therefore, SMC is still the therapy worthy first recommendation, while other non-pharmacologic treatments are also worth trying.

We describe our research strategy, inclusion criteria, and data extraction process in detail. In order to get a reasonable estimate, we used the intention-to-analysis, with all dropouts assumed not having benefit from treatment. Random effects model was used to pool data to reduce the likelihood that any beneficial effect of non-pharmacologic therapies in CIC has been overestimated. Although heterogeneity was high in most of our analyses, it is mostly between different treatment measures. We conducted subgroup analysis based on different clinical outcomes, so we did not perform sensitivity analysis. Although few studies were included, we conducted a network meta-analysis for the first time to compare different non-pharmacologic auxiliary treatments in children with CIC. Biofeedback and physiotherapy are currently the most widely used adjuvant therapies in addition to standard medical care, and they also performed better in this network meta-analysis compared to other methods.

Chronic idiopathic constipation mainly originates from uncoordinated movement of pelvic floor muscles and colonic motility disorder during defecation. Biofeedback therapy uses instruments to record certain biological information related to psychological and physiological activities that the human body is not aware of under normal circumstances (such as myoelectric activity, pressure changes, etc.) and convert it into perceptible “acoustic and optical signals.” Show it to the patient in a “visual and audible” form, allowing them to recognize their abnormal bowel movements. Under the guidance of computer programs and therapists, patients learn to consciously control their own abnormal psychological and physiological activities, gradually correct the uncoordinated movement of pelvic floor muscles and abdominal muscles, and achieve the purpose of curing constipation (28). Therefore, this treatment method is mainly carried out in older children because it requires them to effectively communicate with the therapist and accept this program psychologically and behaviorally to achieve a certain therapeutic effect.

Physiotherapy has been paid more and more attention in recent years. The main principle is to instruct patients to perform defecation-related muscles and sensory training to coordinate defecation. It mainly includes improving toilet behavior and posture, raising the awareness of defecation, learning to relax and increasing intra-abdominal pressure while defecating (29, 30). This program has a significant advantage. It only requires the guidance of experienced physiotherapists and does not require the assistance of relevant equipment, which may help promote it in some developing countries and areas.

There are several limitations in this study. Most studies were conducted in referral populations, which means that the relative efficacy of these treatments is unclear in primary care populations. Because there were no head-to-head comparisons between different auxiliary treatments, estimates of relative effectiveness could only be based on indirect comparisons. There are several excluded RCTs reporting other non-pharmacologic therapies such as reflexology, interferential electrical stimulation. Simultaneously, the control treatments were non-standard medical care or the results could not be transmitted in this network meta-analysis (31, 32). Most of the included studies used curative rate as a primary outcome. The curative rate was calculated based on the symptoms of defecation frequency and fecal incontinence while not address other trouble problems, such as abdominal pain, straining during defecation, a sensation of incomplete evacuation, and feeling of anorectal obstruction. The effects of these auxiliary treatments involved in this meta-analysis on the above symptoms deserve further attention. Currently, the standard medical care for functional constipation contains dietary adjustment, toilet training, reassurance, education, and the use of laxatives. Most of the eligible studies did not describe the detailed drug use information, such as specific type, dosages. It may be because the types of laxatives suitable for children are limited, and all the included RCT studies did not provide detailed information about the use of drugs. The intervention methods were non-pharmacologic auxiliary treatments, which may also be another important reason. Therefore, we also did not consider the difference in clinical outcomes caused by different laxatives. Some clinical trials have compared the therapeutic effects of different laxatives in adults with constipation in recent years (33, 34). No high-quality clinical studies such as RCTs and relevant meta-analysis to compare the effects of the optimal doses of different drugs in pediatric constipation. In the future, we can design relevant RCT studies to clarify the role of different drugs in children with constipation to provide high-quality evidence for clinical decision-making.

Network meta-analysis is able to compare the efficacy of different treatments without direct comparisons to provide credible ranking evidence for helping clinical decisions (21). Although almost all non-pharmacologic therapies are effective complementary therapies for treating pediatric CIC, they are needed to be used with SMC to enhance efficacy. Physiotherapy and biofeedback are the two-better choices among these four non-pharmacologic treatments. These evidence-based recommendations are important for patients, clinicians, and policymakers. In the future, large sample head-to-head trials of non-pharmacologic therapies are needed to obtain higher levels of evidence for the management of CIC children.

## Data Availability Statement

The original contributions presented in the study are included in the article/Supplementary Material, further inquiries can be directed to the corresponding author.

## Author Contributions

All the authors contributed to the work and approved the final version of the manuscript.

## Conflict of Interest

The authors declare that the research was conducted in the absence of any commercial or financial relationships that could be construed as a potential conflict of interest.

## Supplementary Material

Supplementary Figure 1 Risk of bias and summary of all included studies using the Risk of Bias Assessment Tool for Randomized studies. **(A)** Bias graph; **(B)** Bias summary. +: low risk bias; –: high risk of bias; ?: unclear risk of bias.

Supplementary Figure 2 Forest plots of randomized controlled studies of treatments in the management of chronic idiopathic constipation. **(A)** The forest plot and heterogeneity (*I*^2^-values) between trials concerning the endpoint of the absence of constipation with laxatives allowed. **(B)** The forest plot and heterogeneity (*I*^2^-values) between trials with respect to the endpoint of an absence of constipation with laxatives not allowed. **(C)** The forest plot and heterogeneity (*I*^2^-values) between trials with respect to the endpoint of defecation frequency per week with laxatives allowed.
