# Supplementary figures and images for: Efficacy of Non-pharmacologic Auxiliary Treatments in Improving Defecation Function in Children With Chronic Idiopathic Constipation: A Systematic Review and Network Meta-analysis
Source: Front Pediatr. 2021 Apr 27;9:667225. doi: 10.3389/fped.2021.667225 (PMC8110729; doi:10.3389/fped.2021.667225)

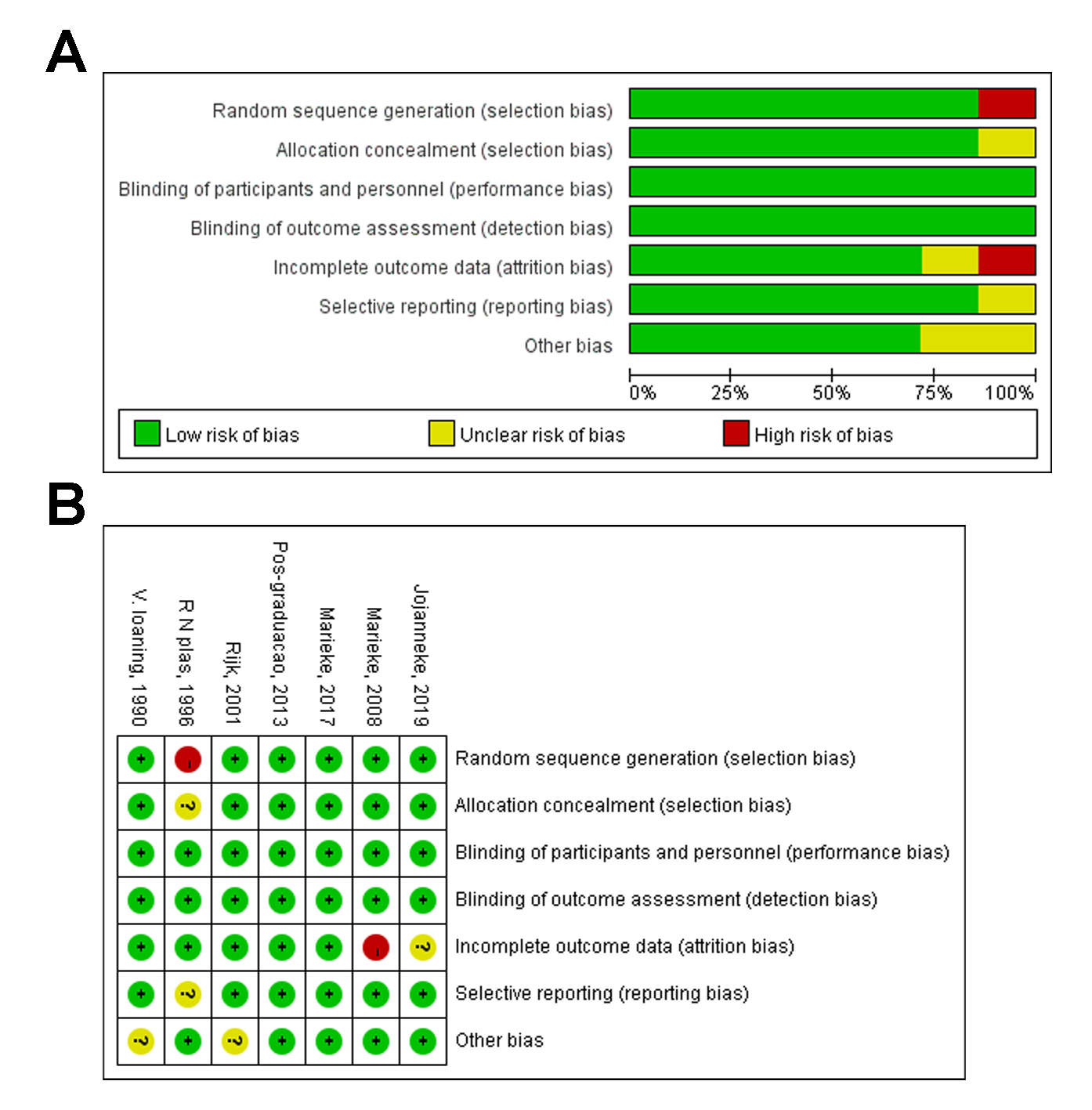

Supplement: Supplementary Figure 1 — Risk of bias and summary of all included studies using the Risk of Bias Assessment Tool for Randomized studies. (A) Bias graph; (B) Bias summary. +: low risk bias; –: high risk of bias; ?: unclear risk of bias. [file Image_1.TIF]

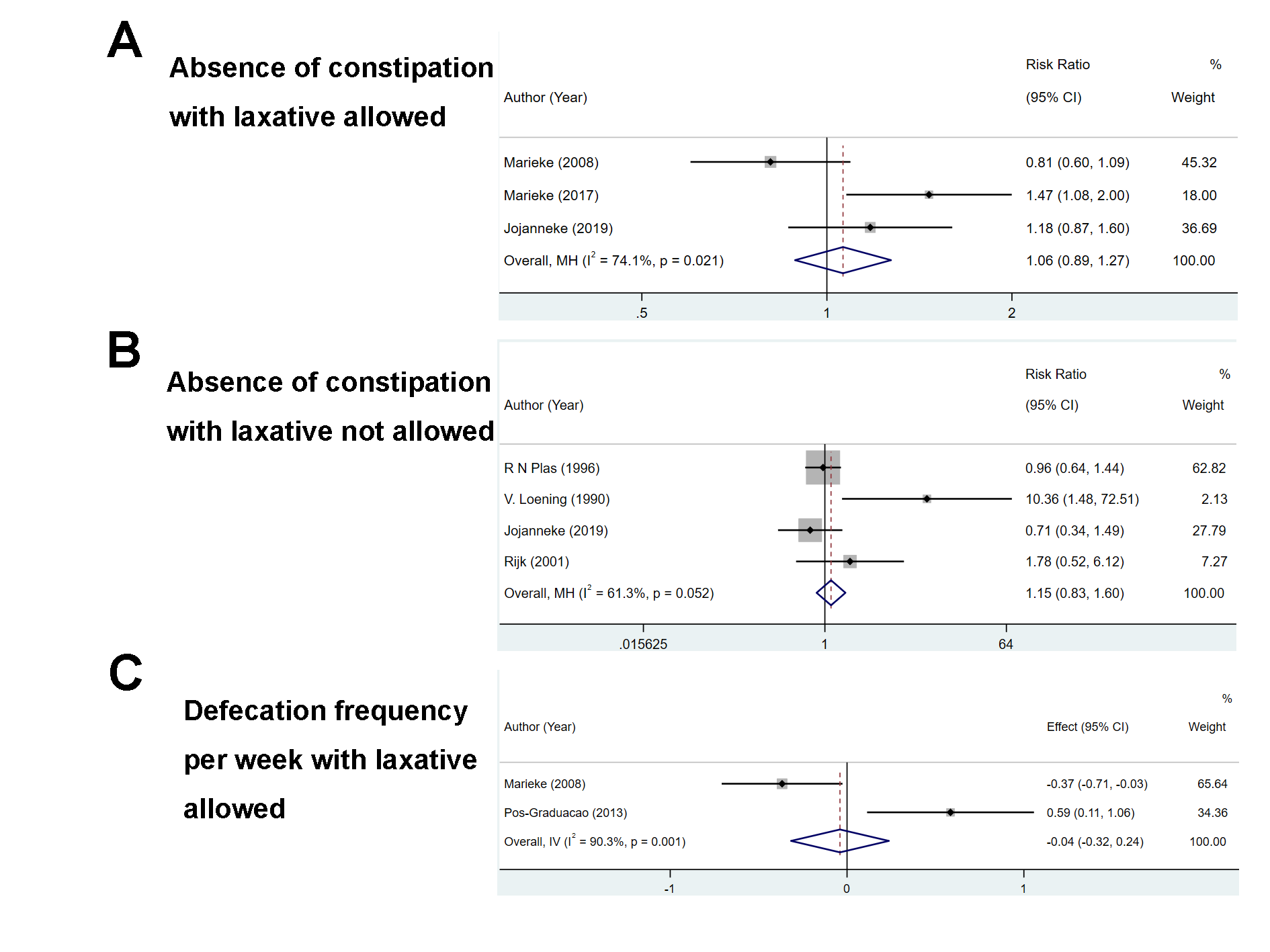

Supplement: Supplementary Figure 2 — Forest plots of randomized controlled studies of treatments in the management of chronic idiopathic constipation. (A) The forest plot and heterogeneity (I2-values) between trials concerning the endpoint of the absence of constipation with laxatives allowed. (B) The forest plot and heterogeneity (I2-values) between trials with respect to the endpoint of an absence of constipation with laxatives not allowed. (C) The forest plot and heterogeneity (I2-values) between trials with respect to the endpoint of defecation frequency per week with laxatives allowed. [file Image_2.TIF]
